# Upregulation of psoriasin/S100A7 correlates with clinical severity in patients with oral lichen planus

**DOI:** 10.1007/s00784-024-05717-z

**Published:** 2024-05-16

**Authors:** Kim Natalie Stolte, Kerstin Danker, Maren Witt, Harald Ebhardt, Henrik Dommisch

**Affiliations:** 1 Department of Periodontology, Oral Medicine and Oral Surgery, Charité - Universitätsmedizin Berlin, corporate member of Freie Universität Berlin, Humboldt-Universität zu Berlin, and Berlin Institute of Health, Assmannshauser Straße 4-6, 14197 Berlin, Germany; 2 Berlin Institute of Health at Charité - Universitätsmedizin Berlin, BIH Biomedical Innovation Academy, BIH Charité Junior Clinician Scientist Program, 10117 Berlin, Germany; 3 Institute of Biochemistry, Charité-Universitätsmedizin Berlin, Corporate Member of Freie Universität Berlin, Humboldt Universität zu Berlin, and Berlin Institute of Health, 10117 Berlin, Germany; 4 Zentrum für Oralpathologie, 14482 Potsdam, Germany

**Keywords:** S100A7, Psoriasin, Oral lichen planus, Inflammation, Antimicrobial peptides, OHIP

## Abstract

**Objectives:**

The aim of this study was to: (1) investigate the expression patterns of antimicrobial peptides (AMPs), specifically psoriasin (S100A7) and calgranulin A and B (S100A8/A9), in patients with oral lichen planus (OLP) compared to healthy individuals; (2) evaluate the oral health-related quality of life (OHrQoL) in OLP patients versus healthy controls; (3) investigate the impact of clinical severity of OLP on OHrQoL; and (4) assess the influence of AMP expression on clinical severity and OHrQoL in OLP patients.

**Materials and methods:**

Oral mucosal biopsies (*n* = 38) were collected from healthy individuals (*n* = 17) and patients with OLP (*n* = 21). Levels of AMPs (S100A7, S100A8, S100A9) and pro-inflammatory cytokines interleukin-8 (IL-8) and tumor necrosis factor alpha (TNFα) were assessed by RT-qPCR. AMP protein localization was identified by indirect immunofluorescence analysis. OHrQoL was assessed using the OHIP-G14 questionnaire, and clinical severity was evaluated with the Oral Disease Severity Score (ODSS). Correlations between OLP manifestation, OHrQoL, and AMP expression were evaluated.

**Results:**

(1) S100A7 (*p* < 0.001), IL-8 (*p* < 0.001), and TNFα (*p* < 0.001) mRNA levels were significantly upregulated in OLP tissue compared to healthy tissue, while S100A8 (*p* < 0.001) and S100A9 (*p* < 0.001) mRNA levels were downregulated. Immunofluorescence staining revealed an enhanced expression of S100A7 and decreased protein expression of S100A9 in OLP tissue. (2) OLP patients (9.58 ± 8.32) reported significantly higher OHIP-G14 scores compared to healthy individuals (0.67 ± 0.87; *p* < 0.001), particularly in the categories “physical pain” (*p* < 0.001) and “psychological discomfort” (*p* = 0.025). (3,4) Clinical severity (25.21 ± 9.77) of OLP correlated positively with OHrQoL (ρ = 0.497) and psoriasin expression (ρ = 0.402).

**Conclusions:**

This study demonstrated differential expression patterns of AMPs in OLP and highlighted the correlation between the clinical manifestation of OLP and OHrQoL. Further research approaches should address the role of psoriasin in the risk of malignant transformation of OLP.

**Clinical relevance:**

Psoriasin is a putative biomarker to monitor disease severity including malignant transformation of OLP lesions. OHIP-G14 scores can be useful to monitor OHrQoL in OLP patients.

## Introduction

Lichen planus is a chronic inflammatory autoimmune disease that can affect the skin, nails, genitourinary tract, and oral mucosa. Oral lichen planus (OLP) clinically manifests as characteristic lesions, including white striae or patches, erosive areas, and ulcers. Symptoms may range from burning sensation to destruction of the epithelial surface with painful sores, limitations in ingestion and articulation, and development of squamous cell carcinoma. The disease has been classified by WHO as a potentially malignant lesion, with a 1.4% risk of malignant transformation [1, 2]. Despite the well-characterized clinical manifestations of OLP, its pathogenesis is still largely unknown. However, an abnormal T cell-mediated immune response leading to apoptosis of basal epithelial cells is suspected. Due to the incomplete understanding of the etiology, there is no curative treatment for OLP. Therapy focuses on symptom relief and usually involves topical corticosteroids. Given the complexity and yet incompletely understood pathogenesis as well as the potential of malignant transformation of OLP lesions, there is an urgent need to explore new approaches for its diagnosis, disease monitoring, and treatment.

Antimicrobial peptides (AMPs) are small proteins synthesized by numerous human cells including oral epithelial cells, and AMPs are part of the innate immune response and directly affect oral microorganisms [3–5]. In humans, several types of AMPs have been identified, including human β-defensins (hBDs), cathelicidin LL-37, some chemokines and S100 proteins [6]. AMPs are expressed in epithelial tissues, odontoblasts of the dental pulp, salivary glands, and can be found in saliva and in gingival crevicular fluid. They function by binding to microbial membranes, forming pores, and disrupting membrane integrity [3, 4, 6]. While some AMPs are constitutively expressed in the oral cavity to maintain homeostasis, others are upregulated during inflammatory conditions [7–10]. Recent studies have also highlighted the role of noncoding RNA molecules, such as microRNAs (miRNAs), in regulating oral tissue homeostasis [11–14]. These miRNAs can potentially influence AMP synthesis and function by targeting their mRNA. Certain miRNAs exhibit differential expression in oral inflammatory and autoimmune disorders, such as OLP, and have been linked to its potential malignant transformation, underscoring their importance in both normal physiology and disease pathogenesis [15].

S100 proteins are calcium-binding proteins that are expressed in various cells of the oral mucosa, including epithelial cells, immune cells, and fibroblasts. They play a crucial role in the oral innate immune response by mediating immune cell recruitment, antimicrobial activity, immune regulation, and tissue repair. Calprotectin is a heterodimer derived from the proteins S100A8 (calgranulin A) and S100A9 (calgranulin B) and is constitutively expressed in cells of stratified oral epithelia and in cultured gingival epithelial cells during health [16]. S100A7, also known as psoriasin, has been extensively studied in the context of psoriasis, a chronic inflammatory skin disease characterized by hyperproliferation and aberrant differentiation of keratinocytes [17]. Furthermore, psoriasin has been found to be overexpressed in epithelial tumors, such as breast cancer and oral squamous cell carcinoma [18, 19]. In light of the fact that OLP is considered an oral potentially malignant lesion, the examination of this AMP seems to be of particular relevance [1].

AMPs with their multiple functions, including antimicrobial activity and mediator-like functions in inflammation, play a key role in innate immunity and also in cancer biology [6, 20]. While hBDs have been studied in OLP, there is, however, a lack of knowledge regarding expression profiles and the role of S100 proteins [21].

The rationale for this study was to gain scientific information on the pathogenesis of OLP in relation to the clinical severity and the oral health-related quality of life (OHrQoL). The underlying mechanisms leading to malignant transformation, and in this context, the potential role of AMPs as putative biomarkers, are yet unclear. Thus, this study aimed at examining expression profiles of AMPs, including S100A7, S100A8, and S100A9 in patients with OLP compared to healthy controls in relation to disease severity and OHrQoL.

We hypothesized that AMPs are differentially expressed in OLP lesions, and in this context, an upregulation of inflammation-induced AMPs such as S100A7 and a downregulation of constitutively expressed AMPs such as S100A8/9 in OLP.

Further, this study aimed at assessing the OHrQoL of patients with OLP and evaluated the correlation between clinical manifestation and OHrQoL, emphasizing the importance of early diagnosis and effective management of this condition. In this context, the key objective was to assess the impact of the AMP expression in correlation to OHrQoL and clinical severity of OLP.

Understanding the role of AMPs may provide insights into pathogenesis and progression, including the malignant transformation, of OLP. The analysis of AMP expression profiles exhibits great potential for the development of novel diagnostic and therapeutic strategies that help to manage and monitor OLP, and therewith, improve patients’ quality of life.

## Materials and methods

### Study population

Patients were enrolled at the Department of Periodontology, Oral Medicine and Oral Surgery, Charité - Universitätsmedizin Berlin. The inclusion criteria encompassed individuals aged between 18 and 85 years, irrespective of gender (m/f/d). For the control group, tissue samples from healthy individuals were obtained during medically indicated oral surgical procedures, such as the extraction of displaced wisdom teeth. In cases where oral lichen planus (OLP) was clinically suspected, as per the criteria defined by the WHO Collaborating Centre (2020), samples of the affected oral mucosa were routinely collected [22]. These samples were then subjected to histopathological examination to confirm the diagnosis of OLP and to exclude epithelial dysplasia.

Exclusion criteria were:

- children and adolescents under 18 years of age;
- pregnant women and lactating mothers;
- present or previous history of oral carcinoma;
- serious psychiatric illness;
- alcoholism;
- severe blood coagulation disorders;
- terminal or serious illness with significant limitation of mobility and overall vitality;
- temporary: ancillary findings of the oral mucosa requiring therapy, e.g. candidiasis.

The study was approved by the Ethics Committee of the Charité - Universitätsmedizin Berlin (IRB number: EA4/057/22), and patients gave their written informed consent.

### Biopsy sampling

Prior to surgical excision, local anesthesia (1mL, UDS; Sanofi, Frankfurt am Main, Germany) was administered to an area approximately 10 mm apart from the sampling area to avoid any effect of the anesthetic. A 4 mm diameter punch biopsy was performed. The area was closed with a suture which was removed after 5–7 days. Depending on the planned examination method, the tissue samples were subsequently stabilized in Allprotect Tissue Reagent (Qiagen, Hilden, Germany) or fixed with 4% buffered formalin. A total of 17 healthy subjects (10 female, 7 male) and 21 patients with OLP (17 female, 4 male) were included.

### Total RNA purification

A maximum of 20 mg of frozen tissue stabilized in Allprotect Tissue Reagent was placed in a 2 ml tube with a sterile stainless-steel ball and crushed in a vibrating mill at 30 Hz for 2 min. Total RNA purification was carried out using an AllPrep® DNA/RNA/miRNA Universal Kit (Qiagen, Hilden, Germany) according to the manufacturer’s instructions. Total RNA concentration was determined using the nanodrop technology (multi-plate reader, Thermo Fisher Scientific, Waltham, USA).

### Quantitative reverse transcription polymerase chain reaction (qRT-PCR)

Gene expression was quantified by quantitative reverse transcription polymerase chain reaction (qRT-PCR), as previously described [23]. Briefly, 500 ng of total RNA was transcribed into cDNA using the High-Capacity cDNA Reverse Transcription Kit (Applied Biosystems) and oligo-(dT)-primers (Thermo Fisher) in full accordance with the manufacturer’s guidelines. Control reactions contained water instead of cDNA. Each biological sample was analyzed in triplicates using the CFX Connect System (Bio-Rad, USA) in combination with SYBR Select Master Mix (Thermo Fisher Scientific). Gene expression of TNFα, IL-8, S100A7, S100A8, and S100A9 was normalized to the mRNA expression of GAPDH, and relative expression was calculated (Microsoft Excel, Redmond, WA, USA; GraphPad Prism Software, La Jolla, CA, USA), using the 2^−ΔΔCT^ method, as previously described [24]. Primer Sequences were as follows:

- GAPDH (forward: TCG TGG AAG GAC TCA TGA CC; reverse: ATG ATG TTC TGG AGA GCC CC).
- IL-8 (forward: AAC TTC TCC ACA ACC CTC TG; reverse: TTG GCA GCC TTC CTG ATT).
- TNFα (forward: CCT GCT GCA CTT TGG AGT GA; reverse: GAG GGT TTG CTA CAA CAT GGG).
- S100A7 (forward: AGA CGT GAT GAC AAG ATT GA; reverse: TGT CCT TTT TCT CAA AGA CGT C).
- S100A8 (forward: CCT CTC AGC CCT GCA TGT CT; reverse: CGG TCA ACA TGA TGC CCA CG).
- S100A9 (forward: TCA TCA ACA CCT TCC ACC AA; reverse: TTA GCC TCG CCA TCA GCA) (metabion GmbH, Planegg/Steinkirchen, Germany).

### Immunohistochemical preparation of paraffin-embedded tissues and haematoxylin-eosin staining

Specimens were immediately fixed in 4% buffered formalin and subsequently embedded in paraffin. Serial histological sections of 4 μm were deparaffinized and rehydrated using graded ethanol and deionized water, respectively. Deparaffined sections were stained with haematoxylin (Carl Roth, Karlsruhe, Germany) and eosin (0.1% in Aqua dest.; Carl Roth) to enable the assessment of the architecture of lichen-affected and healthy tissue.

### Psoriasin and calgranulin B staining

The following antibodies were used in this study: psoriasin/S100A7 (H-8, mouse, monoclonal; sc-377,084; dilution 1:50; Santa Cruz Biotechnology Inc.); Calgranulin B /S100A9(B-5, mouse, monoclonal; sc-376,772; dilution 1:50; Santa Cruz Biotechnology Inc.). Histological sections were incubated with the primary antibody overnight at 4 °C. Antibodies were diluted using 3% BSA in PBS. Subsequently, sections were incubated with the secondary biotinylated anti-mouse IgG antibody (dilution 1:1000, Thermo Fisher). Cell nuclei were stained with Hoechst 33342 dye (Sigma-Aldrich, St. Louis, USA). Sections from a total of three donors were stained simultaneously using the same antibody mix.

### Oral health impact profile (OHIP)

Eligible subjects were asked to complete a questionnaire (OHIP-G14) on oral health-related quality of life (OHrQoL). The OHIP-G14 questionnaire evaluates functional limitation, physical pain and disability, psychological discomfort and disability, social disability, and handicap items. This tool is reliable, valid and precise for assessing the impact of oral health issues on individuals’ lives [25]. The questionnaire comprises 14 items that inquire about the impact of oral health issues experienced during the preceding month. The maximum score is 56 and higher scores indicate a poorer OHrQoL.

### Oral disease severity score

The Oral Disease Severity Score (ODSS) is a disease severity scoring system designed by a consensus of experts and validated for use in pemphigus vulgaris and mucous membrane pemphigoid. It has recently been applied to OLP [26]. The composite score encompasses 17 oral sites and an activity score, enabling an accurate assessment of disease severity. To avoid inter-observer bias, the classification was performed by one observer.

### Statistical analysis

Statistical differences between the two biopsied groups were calculated using Student t-test if there was a normal distribution of the data (IBM SPSS® Statistics Version 29.0.0.0, IBM, Armonk, NY, USA). This was verified using the Shapiro-Wilk test. Otherwise, significant differences were determined by Mann-Whitney-U-test. When indicated, Bonferroni correction was applied for multiple testing.

## Results

### Gene expression profile in human biopsies

**Fig. 1:**
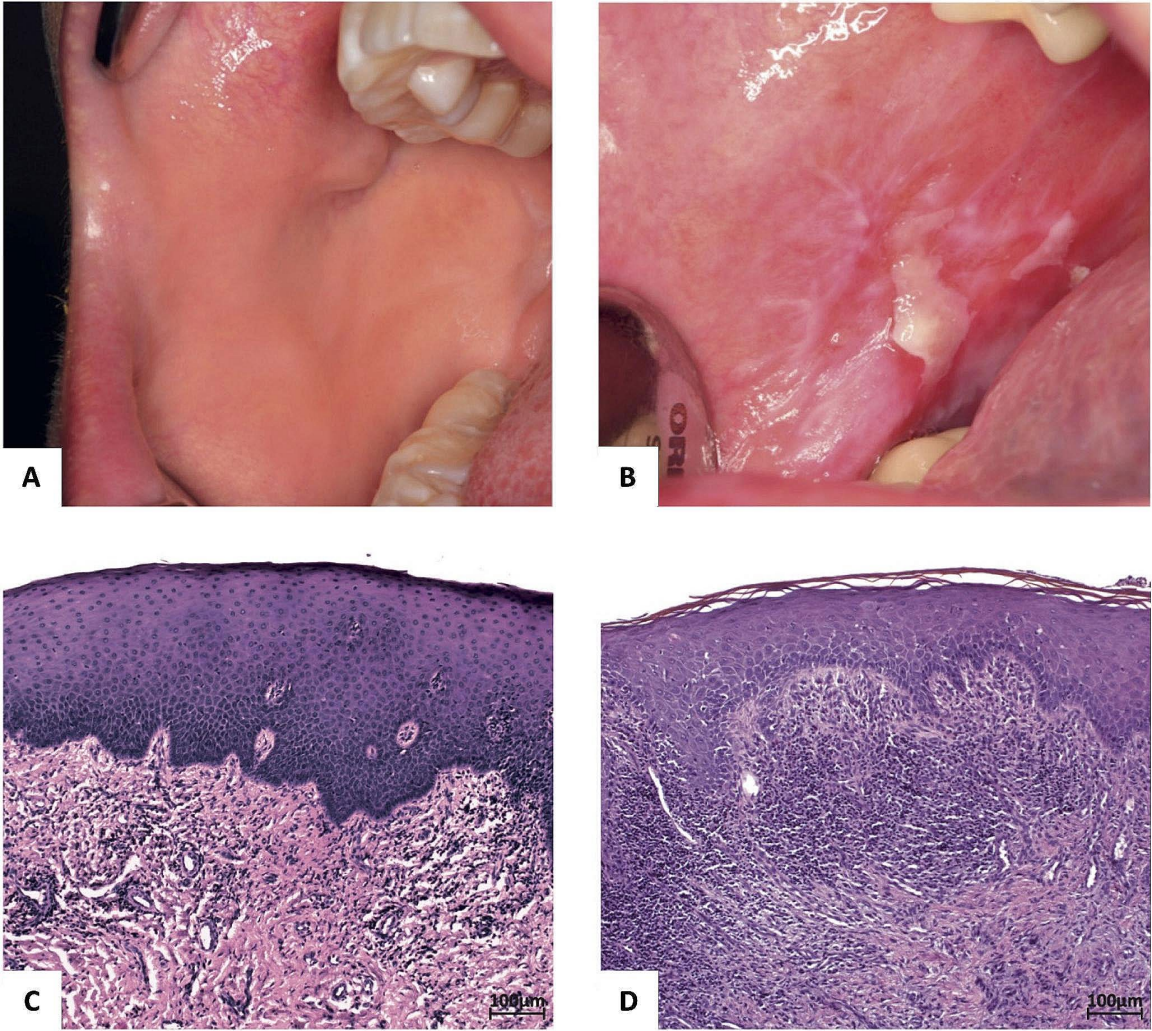
Representative comparison of clinical (**A**, **B**) and histological (**C**, **D**) findings of a healthy subject (**A**, **C**) and OLP patient (**B**, **D**). Photographic representation of the oral mucosa of the *planum buccale dextrum* in the healthy state (**A**) and with OLP. In addition to reticular striae on a reddened base, a central erosion is shown here (**B**). Microscopically, haematoxylin-eosin staining (**C**, **D**) revealed a squamous mucosa with regular stratification of the epithelium by morphologically inconspicuous keratinocytes and subepithelial connective tissue. In OLP (**D**), there is subepithelial band-like lymphocytic inflammation along with some Civatte bodies in the basal epithelial layer. Scale bar 100 μm

We included 17 healthy subjects (10 females, 7 males; age: 33,5 ± 20,7years) and 21 patients with OLP (17 female, 4 male; age:61,8 ± 16,5 years) for genetic analyses (Fig. 1). Three healthy subjects and one patient with OLP were smokers. Thyroid disorders were reported in 9 OLP patients, but only in one healthy participant.

**Fig. 2:**
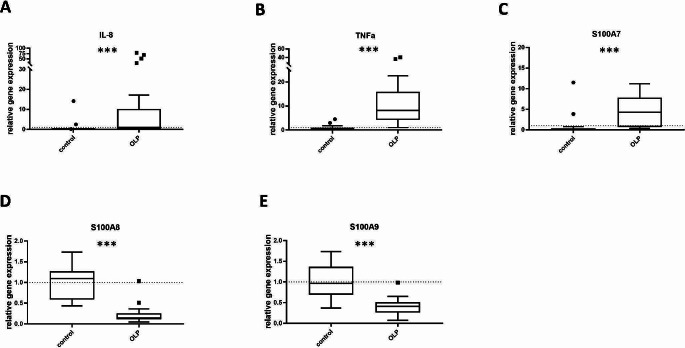
Elevated gene expression of pro-inflammatory cytokines IL-8 (**A**), TNFα (**B**) and S100A7 (**C**) and downregulated gene expression of antimicrobial peptides S100A8 (**D**) and S1009 (**E**) in OLP tissue compared to healthy oral mucosa was observed. Relative gene expression in oral mucosa of healthy subjects (*n* = 17) and patients with OLP (*n* = 21) was determined by qRT-PCR. Gene expression of IL-8, TNFα, S100A7, S100A8 and S100A9 was normalized to the mRNA expression of GAPDH, and relative expression was calculated. *** indicates *p* < 0.001

The gene expression of proinflammatory cytokines (IL-8, TNFα) and AMPs (S100A7, S100A8, S100A9) was assessed in biopsies from healthy controls and patients with OLP (*n* = 38, Fig. 2).

### Upregulation of pro-inflammatory cytokines in oral lichen planus biopsies

Biopsies of patients with histopathologically confirmed OLP showed a significant upregulation of the proinflammatory cytokines IL-8 (Fig. 2A) and TNFα (Fig. 2B; *p* < 0.001, respectively) compared to biopsies derived from healthy tissues.

### Alteration of antimicrobial peptide mRNA levels in oral lichen planus

The mRNA expression of S100A7, Fig. 2C), was significantly increased in OLP compared to the control group (*p* < 0.001,). In contrast, mRNA expressions of S100A8 (Fig. 2D) and S100A9 (Fig. 2E) were downregulated in the OLP group when compared to the control group (*p* < 0.001, respectively).

### Histopathological detection of civatte bodies

Histopathological analysis revealed that OLP samples displayed a squamous mucosa with regular stratification of the epithelium by morphologically inconspicuous keratinocytes. In addition, there was increased parakeratosis of the epithelial surface. Subepithelially, a band-like lymphocytic inflammation with penetration into the epithelium was seen. In some cases (Fig. 1D), there was focal degeneration of the basal epithelium with evidence of Civatte bodies. In OLP biopsies, Civatte bodies were detected in 11 out of 19 samples. Candida infection was excluded using PAS reaction.

### Alteration of antimicrobial peptide protein expression in oral lichen planus revealed by immunofluorescence analyses

**Fig. 3:**
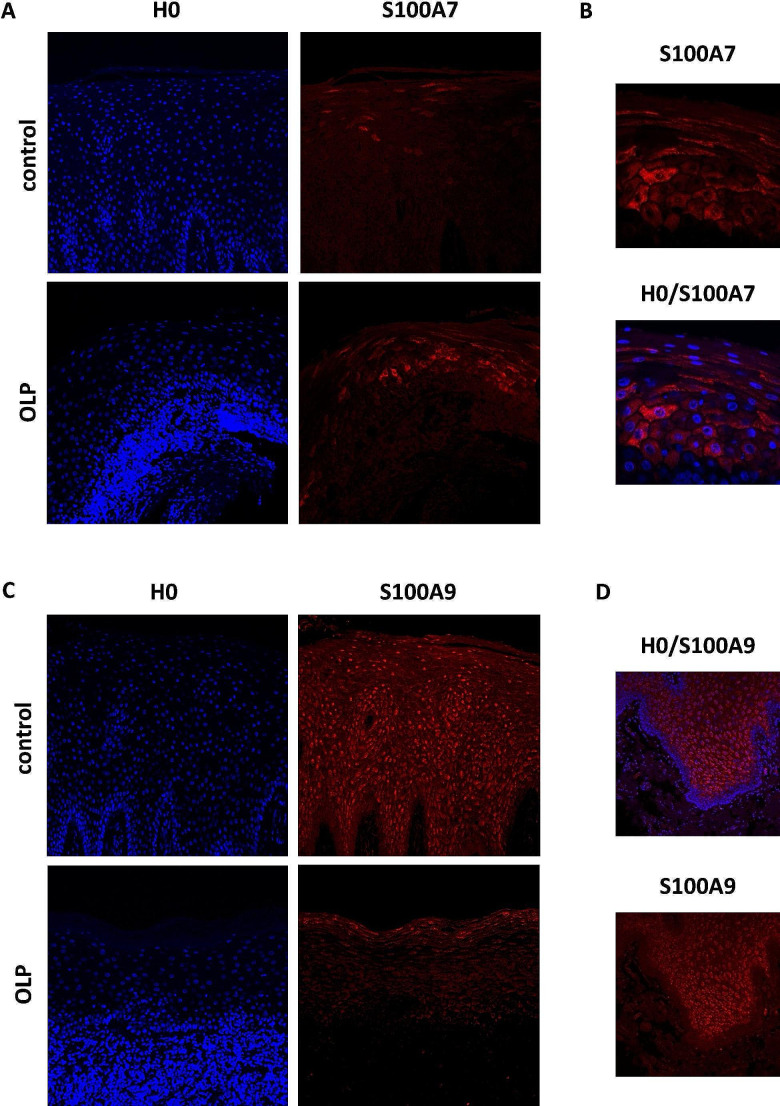
Immunofluorescence analysis of S100A7 and S100A9 (red) in human oral mucosa. Indirect immunofluorescence showed upregulation of S100A7 in OLP tissue compared to healthy controls (**A**, magnification 20x). Histological pattern in OLP revealed a band-like cell infiltrate visualized by nuclear staining H0 (blue). S100A7 showed a cytosolic distribution primarily present in the *stratum spinosum* and *granulosum* (**B**, magnification 63x). In contrast, downregulation of S100A9 in OLP samples was found (**C**) with positive signals across all cell layers except the *stratum basale* in healthy tissue (**D**, magnification 20x)

Sections of human OLP mucosa and healthy oral mucosa were stained by immunofluorescence for S100A7 (psoriasin) and S100A9 (calgranulin B). Nuclear staining was performed using Hoechst staining (Fig. 3). Consistent with the qRT-PCR data, there was increased protein expression of S100A7 in OLP tissue (Fig. 3A). A cytosolic distribution of S100A7 was observed, particularly in the *stratum spinosum* and *granulosum*. S100A7 was not detected in the *stratum basale* (Fig. 3B). S100A9 was localized across all cell layers except the *stratum basale* in healthy tissue (Fig. 3C, D). Also, in line with the mRNA data, there was decreased protein expression of S100A9 in OLP tissue (Fig. 3C). In OLP samples, S100A9 was more prevalent in the differentiated layers.

### Clinical parameters of participating subjects

#### OLP patients showed reduced OHrQoL compared to healthy subjects

**Fig. 4:**
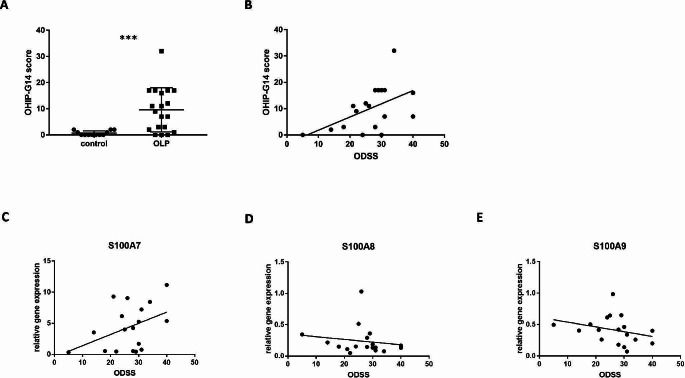
Acquired clinical parameters in relation to the genetic expression of AMPs. Patients with OLP (9.58 ± 8.32) showed higher OHIP-G14 scores compared to healthy subjects (0.67 ± 0.87), reflecting reduced oral health-related quality of life in OLP (**A**). A strong positive correlation (ρ = 0.497) between Oral Disease Severity Score (ODSS) and OHIP-G 14 score was shown (**B**). ODSS increased with increasing gene expression of S100A7 (**C**; ρ = 0.402), indicating a positive correlation. For S100A8 (**D**; ρ=-0.301) and S100A9 (**E**; ρ=-0.485), a negative correlation with the ODSS was found. *** indicates *p* < 0.001

The OHIP-G 14 questionnaire was completed by 31 participants, 12 of whom were in the control group and 19 in the OLP group (Table 1; Fig. 4A). Patients with OLP (9.58 ± 8.32) had a significantly higher OHIP score compared to healthy subjects (0.67 ± 0.87; *p* < 0.001).

**Table 1:** Analysis of responses of healthy subjects (control) and patients with oral lichen planus (OLP) to the Oral Health Impact Profile (OHIP-G14) questionnaire categorized by sub-domains

OHIP-Score (*n* = 31)	control (*n* = 12)		OLP (*n* = 19)		*p*-Value*
	Mean	±SD	Mean	±SD	
Functional limitation	0.00	0.00	0.63	1.05	0.152
*Trouble pronouncing*	0.00	0.00	0.26	0.55	0.346
*Taste worsened*	0.00	0.00	0.37	0.75	0.346
Physical pain	0.33	0.64	3.26	2.13	< 0.001*
*Painful aching*	0.33	0.64	1.63	1.28	0.006*
*Uncomfortable to eat*	0.00	0.00	1.63	1.28	0.001*
Psychological discomfort	0.17	0.38	1.74	1.94	0.025*
*Self-conscious*	0.17	0.38	0.95	1.11	0.093
*Tense*	0.00	0.00	0.79	0.96	0.028*
Physical disability	0.00	0.00	1.26	1.83	0.053
*Diet unsatisfactory*	0.00	0.00	0.63	1.00	0.152
*Interrupt meals*	0.00	0.00	0.63	0.94	0.093
Psychological disability	0.00	0.00	0.89	1.43	0.093
*Difficult to relax*	0.00	0.00	0.53	0.89	0.152
*Embarrassed*	0.00	0.00	0.37	0.75	0.346
Social disability	0.00	0.00	0.89	1.27	0.053
*Irritable*	0.00	0.00	0.53	0.69	0.053
*Difficulty doing jobs*	0.00	0.00	0.37	0.75	0.346
Handicap	0.17	0.38	0.89	1.23	0.120
*Life less satisfying*	0.17	0.38	0.68	0.99	0.205
*Unable to function*	0.00	0.00	0.21	0.53	0.484
Total	0.67	0.87	9.58	8.32	< 0.001*

In particular, there were significant differences especially related to physical pain (*p* < 0.001). OLP patients stated higher scores in painful aching (*p* = 0.006) and in being uncomfortable to eat (*p* = 0.001). In addition, OLP patients reported higher scores in the category of psychological discomfort (*p* = 0.025), being more likely to report being tense (*p* = 0.028).

No significant differences in OHIP-G14 scores (*p* = 0.728) were found between the OLP-group with histopathologically detectable Civatte bodies and the OLP-group without (data not shown).

#### Oral disease severity score correlates with OHIP-G14 scores in OLP patients

The Oral Disease Severity Score (ODSS) is a clinical score used to assess the severity of OLP [26]. Localization of the lesions and an activity score were recorded. The trend was observed that as ODSS rose, OHIP-G 14 score also increased. The Spearman correlation coefficient ρ was 0.497, indicating a strong positive correlation (Fig. 4B). The ODSS in OLP patients was 25.21 ± 9.77 with a mean of 10.47 ± 3.80 for ODSS site and 14.74 ± 6.33 for ODSS activity (Table 2).

**Table 2:** ODSS in OLP patients was 25.21 ± 9.77 (*n* = 19). ^1^ The sites were the outer/inner lips, buccal mucosae right/left, soft palate right/left, hard palate right/left, dorsum of tongue right/left, ventrolateral tongue right/left, floor of mouth right/left, oropharynx right/left and the gingivae (divided into 6 segments). Site score 0 (no lesion) or 1 (lesion), buccal mucosa: 1 (≤ 50%) or 2 (> 50%); dorsum of tongue, floor of mouth, hard or soft palate or oropharynx: 1 (unilateral) or 2 (bilateral) [26]. ^2^ Activity score: 1, mild erythema; 2, marked erythema without ulceration; 3, erosion or ulceration [26]. ^3^ Total Score = Site Score + Activity Score (maximum 96). ^4^ Adapted from [26]

Scoring System	Range	Mean	± SD
ODSS Site^1,4^	2–17	10.47	3.80
ODSS Activity^2,4^	1–26	14.74	6.33
**ODSS Total** ^3,4^	3–40	25.21	9.77

No significant differences in ODSS (*p* = 0.422) were found between the OLP-group with histopathologically detectable Civatte bodies and the OLP-group without (data not shown).

#### Correlation of antimicrobial peptide gene expression and clinical parameters

Correlations between AMP expression and OHIP-G 14 score were tested. Here, no (S100A8, ρ = 0.053) or weak (S100A7, ρ = 0.144; S100A9, ρ = 0.131; *n* = 18) correlations were found between the expression of the AMPs and the OHIP-G 14 score (data not shown).

Increasing ODSS was coherent with an increase in gene expression of S100A7 (Fig. 4C, ρ = 0.402). For S100A8 (Fig. 4D) and S100A9 (Fig. 4E), there was an inverse trend; as gene expression decreased, ODSS increased.

## Discussion

OLP is a chronic inflammatory mucosal disease characterized by mucosal erosions, ulcers, and/or white keratotic lesions and has been classified by the WHO as a potentially malignant lesion [1]. Despite the high prevalence of OLP, there is currently no curative treatment available for affected individuals. The management of OLP typically involves corticosteroids. However, the recurrence rate of OLP after treatment discontinuation, remains high, indicating the urgent need for further research to elucidate the underlying mechanisms to provide more effective and sustainable diagnostic and therapeutic options as well as sufficient clinical parameters to monitor OHrQoL of affected patients.

### Chemical barrier homeostasis is altered in oral lichen planus

The oral cavity is constantly exposed to a range of different microorganisms, but the host maintains a healthy homeostasis through a balanced interplay between microbial colonization and immune defense mechanisms. The oral mucosa provides a primary physical barrier, which is achieved by a high degree of differentiation and cell-cell linkages of the epithelial cells, known as tight junctions (TJ). Studies on oral epithelia have shown that pro-inflammatory cytokines can alter the physical barrier, such as specific expression of TJ proteins [27]. Moreover, alterations of the epithelial barrier with disturbances in desmosomes, adherens and tight junctions in OLP have been reported [28]. In addition to this physical function, the oral epithelium also provides a chemical barrier in the form of antimicrobial peptides (AMPs) as part of the innate immune system [6]. While some AMPs are constitutively expressed in the oral cavity to maintain homeostasis, others are upregulated during inflammatory conditions to protect against pathogens. The human beta-defensin (hBD) -1 is constitutively expressed, whereas hBD-2 and hBD-3 are upregulated in inflamed skin and other epithelia [7–10]. Altered expression of AMPs has been observed in OLP lesions, suggesting a protective function against infection and promoting rapid wound healing [29]. The upregulation of specific AMPs can therefore be interpreted as an intrinsic reaction in the sense of a functioning immune defense system. Although the pathogenesis of OLP is still not fully elucidated, it is thought to be triggered by an altered immune response against oral epithelial tissues, leading to the release of various cytokines and chemokines and thus to chronic inflammation.

### Upregulation of pro-inflammatory cytokines in oral lichen planus biopsies

In addition to the clinical presentation of inflammation documented by ODSS, our data showed increased inflammatory status during OLP compared to healthy status at the mRNA level. This is reflected in the upregulated gene expression of the pro-inflammatory cytokines IL-8 (13-fold) and TNFα (12-fold). These findings are in line with the results of other studies showing significantly higher TNFα, IL-1β and IL-8 levels in OLP saliva compared to saliva of a healthy group [30].

### Altered expression of antimicrobial peptides in oral lichen planus biopsies

Only few studies have investigated the expression and distribution of AMPs in patients with OLP compared to healthy individuals, and of these, most have examined the distribution of hBDs. For example, Salem et al. (2019) found that the expression of human beta-defensin (hBD)-2 was highly induced in OLP lesions compared to controls [8]. hBD-2 and hBD-3 are known to be upregulated in inflamed skin and other epithelia [7]. The aim of this study was to focus on the expression of S100 proteins, as these AMPs play a role in the innate immune response in the oral cavity and have also been associated with malignant epithelial transformation [3, 18]. Therefore, it is essential to investigate their role in oral lichen planus. Although often restricted to the oral cavity, lichen disease is not limited to the mouth, but can also occur on other mucous membrane sites or on the skin. Gambichler et al. (2009) analyzed AMP expression in genital lichen sclerosus (LS) lesions compared to healthy genital skin. They observed significantly higher median mRNA levels of hBD-2, hBD-3 and psoriasin in LS than in controls [21]. These observations derived from genital skin biopsies are consistent with our hypothesis in oral lichen planus and suggest upregulation of inducible AMPs, such as hBD-2 or psoriasin, and downregulation of constitutively expressed AMPs, such as calgranulin B.

These findings suggest that epithelial cells of the oral mucosa respond to a potential lack of epithelial tissue integration as a consequence of OLP. The mRNA of S100A8 and S100A9 is highly expressed in gingival epithelial cells compared to mRNA of e.g. hBD-2 in healthy mucosa [31]. In accordance with the stated hypothesis, downregulation of S100A8 and S100A9 in OLP compared to the control group was observed, suggesting an impaired chemical barrier. Other studies stated that downregulation of S100A8/A9 correlated strongly with a loss of cell cycle control and increased growth of carcinoma cells, which is of special interest since OLP is considered an oral potentially malignant lesion. Therefore, cell dysplasia or transformative changes within the tissue samples examined were excluded by histological analysis by a certified oral pathologist.

Also, consistent with this approach, biopsy analysis revealed a significant increase in S100A7 (psoriasin). To the best of the authors’ knowledge, the expression of psoriasin in oral lichen planus has not yet been assessed in correlation to clinical severity of the disease. Psoriasin had initially been identified as being upregulated in skin lesions of psoriasis patients [17]. In addition, psoriasin has been detected in epithelial malignancies such as skin malignancies [32] and ductal in situ breast carcinomas [18], where high levels of psoriasin correlated with clinical outcomes [33]. Few studies have focused on psoriasin expression in oral pre-malignancies or malignancies. Here, Banerjee et al. found psoriasin to be one of the most intensely up-regulated genes in oral premalignant tissues [34].

Kesting et al. analyzed psoriasin expression in oral squamous cell carcinoma (OSCC) and observed an increased expression at the mRNA level in OSCC samples compared to healthy controls. In the same study, significantly higher levels of psoriasin were found in well-differentiated tumor samples compared to less well-differentiated carcinomas [19], indicating a possible involvement of psoriasin in innate host defense and immune response capacity, suggesting a link between upregulation of psoriasin and early stages of malignant progression. This is particularly relevant when considering OLP as an oral potentially malignant lesion, as it has been classified by the WHO [1].

Several studies have recently identified the significant role of microRNAs (miRNAs) in maintaining oral tissue homeostasis and their altered expression in oral inflammatory and autoimmune disorders such as OLP [11–14]. MiRNAs are small, single-stranded noncoding RNA molecules that regulate protein expression post-transcriptionally, typically by binding to the mRNA 3′ UTR [13]. MiRNAs regulate protein expression primarily post-transcriptionally (Li et al., 2022). Altered miRNA expression has been linked to the potential malignant transformation in OLP [15]. MiRNAs could influence AMPs expression either directly via translational repression or mRNA degradation. By modulating inflammation pathways, miRNAs could also indirectly affect AMP production, e.g. by suppressing pro-inflammatory cytokines, which in turn impacts cytokine-induced AMP expression. Future research approaches could elucidate how miRNAs regulate the expression of AMPs and provide deeper insights into their roles in diseases such as OLP.

The upregulation of psoriasin in OLP tissue was not only shown on mRNA level but also on protein level via indirect immunofluorescence analysis. Psoriasin immunoreactivity of healthy oral mucosa showed scattered expression in the *stratum granulosum* and, to a lesser extent, positive signals in the *stratum corneum* and the uppermost *stratum spinosum*. This is similar to other findings in healthy oral mucosa [19] or healthy genital mucosa [21]. Compared to healthy oral mucosa, psoriasin is highly expressed in OLP. Here, psoriasin was detected in the entire epithelium except for the *stratum basale*. This extended psoriasin localization in the epithelium as well as the upregulation of psoriasin mRNA levels (5-fold) in OLP are consistent with observations of Gambichler et al. on genital mucosa with lichen sclerosus [21].

The clinical severity, documented in this study by the assessment of ODSS, rose with increasing gene expression of psoriasin. Thus, this study provides the first clinical evidence of a significant association between altered psoriasin expression and OLP severity. Previous research on this topic has not focused on quantitative analysis of expression of psoriasin in correlation to clinical features.

In this context, the upregulation of psoriasin may be interpreted as an innate immune reaction by the mucosal epithelial tissue to efficiently combat invading microorganisms due to the impaired epithelial barrier in OLP patients. The degree to which this system functions, or at what point the system tips, remains yet unclear.

The differential expression of AMPs in oral lichen planus patients, characterized by reduced levels of S100A8 and S100A9 and increased levels of S100A7, suggests alterations in the chemical barrier that may enhance susceptibility to oral infections and inflammation. The elevated expression of S100A7, a biomarker previously linked to malignant transformation in skin, breast tissues, and oral mucosa, points to its potential role in OLP disease progression and possibly as an indicator of malignant transformation, though further prospective studies are warranted.

### Clinical features

#### OHrQoL expressed by total OHIP G-14 scores and individual domain responses

The OHIP-G14 questionnaire was chosen to assess oral health-related quality of life (OHrQoL) in OLP patients due to its frequent use in clinical trials, ease of use, and reliability. Being available in multiple languages and preferred by clinicians, it serves as a suitable option in the absence of a definitive selection of Patient Reported Outcome Measures (PROMs) for OLP [35]. Data indicated significantly poorer OHrQoL in patients with OLP compared to healthy individuals. This result was in line with a systematic review and meta-analysis by Yuwanati et al. (2021) showing how OLP negatively affects patients’ OHrQoL [35].

The OHIP-subdomains “physical pain” and “psychological discomfort” of OLP patients were most clearly affected in other studies, which was in line with our findings [36]. In the present study, patients reported significantly higher scores for “physical pain” in particular, “painful aching” and being “uncomfortable to eat”, and significantly higher scores for “psychological discomfort”, i.e. participants reported being more “tense”. In this context, it might be useful to discuss the role of psychological support as part of the therapy. Another study reported that the highest values were reached for the individual questions “painful aching” and “uncomfortable to eat” [37], which also corresponded to our observed maximum values after domain analysis. This questionnaire seemed to reflect the psychological and physical limitations of patients with OLP well and could therefore be used to assess the treatment response and to establish treatment endpoints.

#### Correlation of OHrQoL and clinical assessment

Few studies investigated the reported OHIP scores in relation to the clinical classification of OLP. For instance, other authors found significant differences in OHIP scores between erosive or ulcerative forms and healthy individuals, whereas no significant differences were found between the reticular form of OLP and healthy individuals [36]. In another study, patients with reticular OLP had significantly lower OHIP values than patients with other forms of OLP [37].

According to the authors’ opinion, the identification of a distinct subclass of OLP in everyday clinical practice is often challenging due to the mixed presentation of the disease. As a result, the authors utilized the Oral Disease Severity Score (ODSS) in this study, which not only considers a degree of activity, but also their respective localization of the lesions. The implementation of the ODSS in this study may provide a more comprehensive assessment of OLP severity, leading to a better understanding of its clinical manifestation. In this regard, a strong positive correlation between ODSS and OHIP score was found. Thus, there was a clear correlation between clinical manifestation and quality of life of affected individuals, and the more pronounced the clinical manifestation, the lower the OHrQoL. These findings highlight the negative impact of OLP on patients’ OHrQoL and emphasize the importance of early diagnosis and effective management of the condition.

### Limitations

The findings of this study are based on a limited sample size and restricted to patients from one dental clinic, which may limit the generalizability of our findings. Further, only one oral health-related quality of life (OHrQoL) index was used at one time-point, and therefore a potential change in OHrQoL following clinical response to treatment could not be documented. Therefore, future studies should include larger sample sizes and investigate the therapeutic effects of treatment using multiple OHrQoL indices. To account for the subjective parameter “pain”, a Visual Analogue Scale (VAS) can be included in the documentation in the future. In addition, only a selected set of AMPs was investigated based on the initial hypothesis. For a more global overview and to gain more detailed knowledge of the distribution and regulation of AMPs in OLP, the investigation of additional AMPs as well as their regulation via specific pathways is envisioned for future research.

## Conclusions

Among other functions, AMPs play an important role in the endogenous, non-specific immune defense of the oral epithelium. For the first time, this study showed that the expression of psoriasin, which has been proposed as an indicator of malignant alterations, is increased in oral mucosa of OLP patients compared to healthy individuals and its upregulation was found to correlate significantly with the clinical severity of OLP and adversely impact on OHrQoL. The enhanced expression of psoriasin suggests its role in OLP pathogenesis and its role as a putative biomarker for monitoring disease progression and/or therapeutic response. These findings underscore the need for future investigations identifying the regulatory mechanisms leading to psoriasin or AMP expression, e.g. through specific miRNAs.

Furthermore, this study showed that OHrQoL was reduced in patients with OLP compared to healthy individuals. For the first time, a strong positive correlation between ODSS and OHIP-G14 was demonstrated. The clinical implementation of the investigated target parameters could be discussed with regard to a potential endpoint of symptomatically oriented therapy. The burden of suffering for those affected indicates an increased need for further research to better understand the underlying pathomechanisms of OLP. These findings could contribute to a better understanding of the disease development and help to build new avenues for novel diagnostic and monitoring approaches as well as for preventive and therapeutic interventions.

## Data Availability

Raw data for biopsies are not publicly available to preserve individuals’ privacy under the European General Data Protection Regulation.
